# Allyl Isothiocyanate that Induces GST and UGT Expression Confers Oxidative Stress Resistance on *C. elegans*, as Demonstrated by Nematode Biosensor

**DOI:** 10.1371/journal.pone.0009267

**Published:** 2010-02-17

**Authors:** Koichi Hasegawa, Satsuki Miwa, Kaname Tsutsumiuchi, Johji Miwa

**Affiliations:** College of Bioscience and Biotechnology, Chubu University, Kasugai, Aichi, Japan; Emory Unviersity, United States of America

## Abstract

**Background:**

Electrophilic xenobiotics and endogenous products from oxidative stresses induce the glutathione S-transferases (GSTs), which form a large family within the phase II enzymes over both animal and plant kingdoms. The GSTs thus induced in turn detoxify these external as well as internal stresses. Because these stresses are often linked to ageing and damage to health, the induction of phase II enzymes without causing adverse effects would be beneficial in slowing down ageing and keeping healthy conditions.

**Methodology/Principal Findings:**

We have tested this hypothesis by choosing allyl isothiocyanate (AITC), a functional ingredient in *wasabi*, as a candidate food ingredient that induces GSTs without causing adverse effects on animals' lives. To monitor the GST induction, we constructed a *gst::gfp* fusion gene and used it to transform *Caenorhabditis elegans* for use as a nematode biosensor. With the nematode biosensor, we found that AITC induced GST expression and conferred tolerance on the nematode against various oxidative stresses. We also present evidence that the transcription factor SKN-1 is involved in regulating the GST expression induced by AITC.

**Conclusions/Significance:**

We show the applicability of the nematode biosensor for discovering and evaluating functional food substances and chemicals that would provide anti-ageing or healthful benefits.

## Introduction

The free-living soil nematode *Caenorhabditis elegans* has become one of the most powerful biological research tools available today because of its ease of handling, short lifespan, large brood size, and amenability to molecular as well as classical genetics [1]. Recent studies have revealed that this nematode possesses not only innate defense mechanisms against infection by bacteria and fungi [2]–[4] but also detoxifying systems against xenobiotics [5]. Large numbers of genes in the *C. elegans* genome encode detoxification enzymes such as cytochrome P450 (CYP), short-chain type dehydrogenase (SDR), UDP-glucuronosyl/glucosyl transferase (UGT), and glutathione S-transferase (GST) (WormBase, http://www.wormbase.org/), and they are probably regulated by conserved signal transduction pathways.

Oxidative stresses derived from exogenous and endogenous substances are considered to cause cancer, atherosclerosis, inflammation, hypertension, diabetes, and several age-related diseases [6]. The phase II detoxification enzymes such as GST, glutathione reductase, glutathione peroxidase, UGT, and NAD(P)H:quinone oxidoreductase are recognized as playing major protective roles against oxidants [7]–[9]. Therefore, the induction of phase II enzymes without adverse effects may be one good strategy to reduce the risks of contracting cancer and age-related diseases. Diet-derived inducers of phase II enzymes, such as the isothiocyanates sulforaphane and allyl-isothiocyanate, have been reported as candidates for effective chemopreventives of diseases [10]–[13].

To take advantage of the nematode's defense systems for sensing and responding to xenobiotics, we constructed nematode biosensors, each transgenic animal containing a fusion gene of a phase II enzyme gene with the green fluorescence protein (GFP) gene. We previously reported that with such biosensors we could not only identify toxicants in foods and the environment, but also screen for foods or their ingredients, known or unknown, that would reduce their harmfulness or action [14], [15]. We here report the applicability of our nematode biosensors for the discovery and evaluation of functional food ingredients that serve as anti-ageing agents or protectives against health afflictions, by using allyl isothiocyanate (AITC) as a model functional food ingredient.

## Results

### Nematode Biosensor Detects *gst*-Inducing Activity in Chemicals

**Figure 1:**
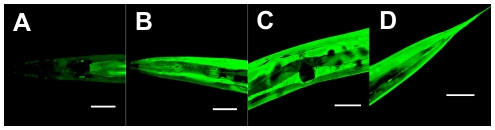
Expression patterns in MJCU049 {*kIs48 [gst-4p::gfp, pDP#MM016B]* V}. Photographs were taken by laser-scanned fluorescence microscopy. (A) Control without acrylamide. Weak GFP signal was detected from hypodermis, body wall muscle, and intestine. (B, C, D) With 500 mg/L acrylamide. Strong GFP signals were detected from the whole body. (B) Head region. (C) Vulval region. (D) Tail region. Scale bars, 50 µm.

Among all of the known *gst* genes (WormBase, http://www.wormbase.org/), *gst-4*, which encodes a sigma class GST, was one of the most up-regulated genes and most prominently expressed when the nematode was treated with the harmful food substance acrylamide [15]. A transcriptional reporter gene (promoter-driven *gfp* transcription) constructed for the *gst-4* promoter (*gst-4p*) was introduced into *C. elegans* to obtain the transgenic nematode MJCU032 {*kEx32 [gst-4p::gfp, pDP#MM016B]*}. Because an extrachromosomal (*Ex*) line sometimes shows unstable mosaic expression, we constructed the chromosomally-integrated line MJCU049 {*kIs48 [gst-4p::gfp, pDP#MM016B]* V} as a nematode biosensor. Its expression pattern did not differ from that of the *Ex* line and was very stable. We confirmed that it showed a typical response against a GST inducer; that is, the nematode biosensor MJCU049 constitutively emitted a residual dim amount of the GFP signal when it was grown on NGM plates and increased intensities when grown on NGM plates containing the GST inducer acrylamide (Figure 1).

**Figure 2:**
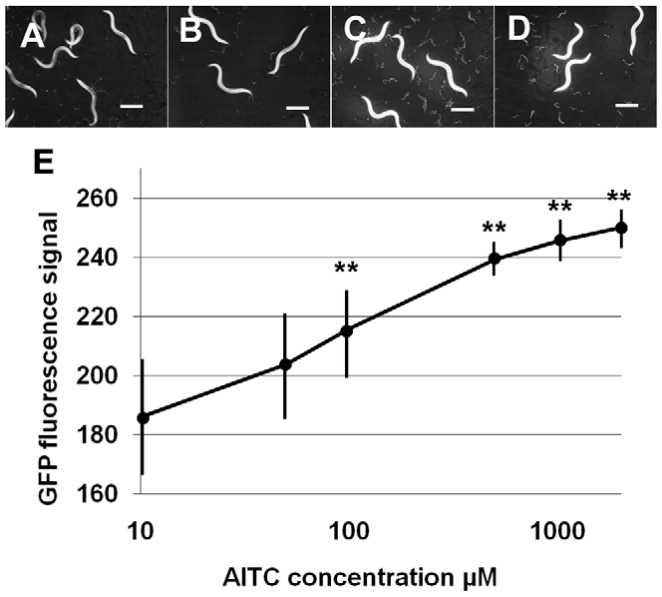
Fluorescence images of MJCU049 exposed to each AITC concentration. (A) Control 0 µM AITC. (B) 100 µM AITC. (C) 1 mM AITC. (D) 2 mM AITC. (E) GFP fluorescence signals were measured and plotted. GFP signals increased at concentrations of more than 100 µM (Kruskal-Wallis test, ** *p*<0.005) in a dose-dependent manner. Scale bars, 500 µm.

Next we used the software FL 1.0.0.1 to analyze the GFP signal kinetics of the nematode biosensor in response to AITC. Figure 2 shows the GFP fluorescence kinetics of MJCU049 in response to AITC exposure at concentrations from 10 µM to 2 mM. Graphs indicate the mean values ± S.E. (N = 23 to 31) for GFP fluorescence signals at the various AITC concentrations from two independent experiments. The GFP fluorescence signal reached the statistically significant strength at or over 100 µM AITC (Figure 2). All nematodes in every AITC concentration were alive 24 hours after exposure to AITC.

### AITC Effects on *C. elegans* Lifespan

Because AITC induced GST-4 expression at concentrations over 100 µM, we next analyzed the effects of AITC on the *C. elegans* lifespan. We treated the nematodes with various AITC concentrations (10 µM to 2 mM) from just-hatched L1 stage (L1 hatchee) or from L4 stage and measured lifespan at 25°C. The mean lifespan of nematodes grown on control plates (without AITC) was 11.4 days, and no adverse effects on the mean lifespan were observed at AITC concentrations of up to 1 mM. But the mean lifespan was significantly reduced when the nematodes were treated with 2 mM AITC from L1 (10.8 days, *P*<0.05) and L4 (10.3 days, *P*<0.005) stage (Table 1).

**Table 1:** Effect of AITC on *C. elegans* lifespan at 25°C.

Concentration	Average ± SEM	Repeat	Med	Max	% difference^a^	*P* value^b^	Total nematode
0 µM	11.4±0.13	4	11	19			394
10 µM L4-	11.1±0.20	2	11	17	−2.13	0.269	116
10 µM L1-	11.0±0.21	2	11	18	−3.42	0.139	117
50 µM L4-	11.3±0.27	2	11	19	−0.50	0.980	98
50 µM L1-	11.6±0.23	2	12	17	+1.89	0.713	109
100 µM L4-	11.4±0.21	3	11	21	+0.05	0.693	187
100 µM L1-	11.3±0.20	3	11	19	−0.34	0.965	179
500 µM L4-	11.0±0.20	3	11	20	−3.09	0.298	174
500 µM L1-	10.8±0.20	3	11	20	−5.36	0.052	175
1 mM L4-	11.1±0.20	3	11	18	−2.72	0.484	186
1 mM L1-	10.8±0.20	3	10	19	−4.92	0.067	168
2 mM L4-	10.3±0.15	4	10	17	−9.51	0.000	255
2 mM L1-	10.8±0.16	4	10	19	−5.21	0.018	209

L1- or L4- after the concentration unit µM or mM indicates that nematodes were treated with AITC from either L1 or L4 stage on, respectively.

a, Percent differences of the mean lifespan compared with that of the control.

b, P values were calculated by log-rank test (vs 0 µM).

### AITC Effect on *C. elegans* Fecundity

**Figure 3:**
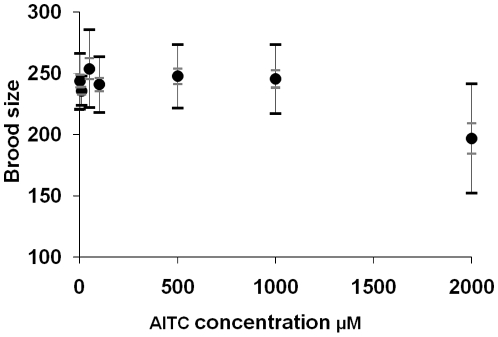
AITC effects on the brood size of *C. elegans* grown at 20°C. All viable progeny of each nematode parent were counted. The averages ± SEM (inner bars) and SD (outer bars) are shown. The significant differences in brood size were determined by Kruskal-Wallis test (** *p*<0.005).

The effects of AITC on the nematodes' reproduction were analyzed by measuring the brood size of the wild-type N2. We treated nematodes from L1 hatchees with various AITC concentrations (0 µM and 10 µM to 2 mM). We did not detect the toxic effects of AITC until 2 mM at which concentration the brood size started to decrease (*P*<0.05, Figure 3).

### AITC Confers Resistance on *C. elegans* against Oxidative Stresses

**Figure 4:**
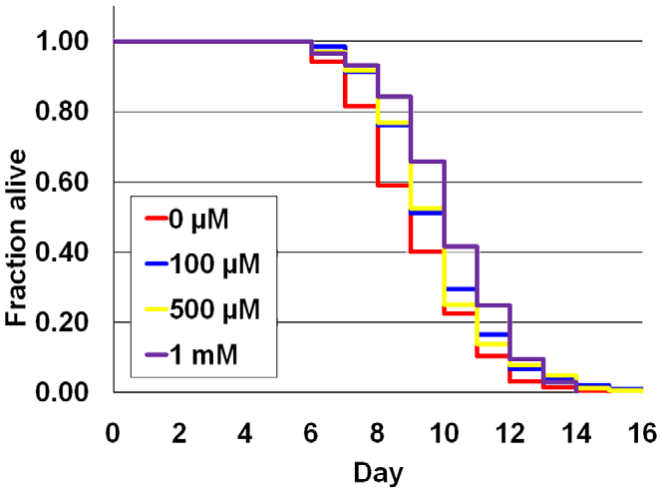
AITC increases resistance of nematodes against 10 mM paraquat. Compared with nematodes without AITC treatment, lifespan was extended significantly when treated with AITC by 6.98% at 100 µM, by 6.38% at 500 µM, and 11.6% at 1 mM. Survival curves are presented based on three individual experiments.

AITC induced GST-4 expression and did not have toxic effects on the nematodes at concentrations from 100 µM to 1 mM. Next, we analyzed the effects of AITC on *C. elegans* lifespan under oxidative stress conditions. Nematodes from L1 hatchees were treated for five days with AITC (0 µM, 100 µM, 500 µM, and 1 mM) before being transferred onto the NGM plates containing 10 mM paraquat, and then their lifespan was measured. When the nematodes were grown on 10 mM paraquat, the mean lifespan was 9.1 days, but the mean lifespan was extended significantly by treatment with AITC; that is, it increased by 6.98% (p<0.05) at 100 µM, 6.38% (p<0.05) at 500 µM, and 11.6% (p<0.001) at 1 mM (Figure 4, Table 2).

**Table 2:** Protective effect of AITC on *C. elegans* against paraquat.

Concentration	Average ± SEM	Repeat	Med	Max	% difference^a^	*p* value^b^	Total nematode
0 µM AITC	9.13±0.12	3	9	15		222	
100 µM AITC	9.76±0.13	3	10	16	+6.98%	0.011	194
500 µM AITC	9.71±0.14	3	10	16	+6.38%	0.027	168
1 mM AITC	10.19±0.20	3	10	14	+11.6%	0.000	178

a, Percent differences of the mean lifespan compared with that of the 0 µM AITC.

b, P values were calculated by log-rank test (compared with 0 µM AITC).

**Figure 5:**
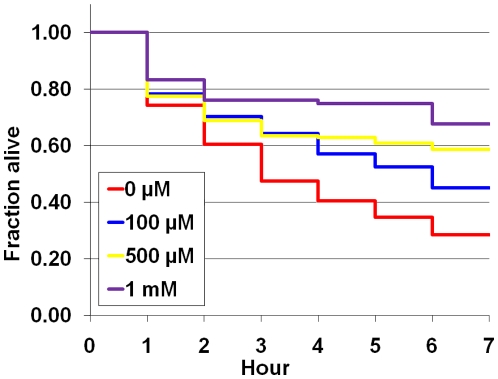
AITC increases resistance of *C. elegans* against 200 µM juglone. Compared with nematodes without AITC treatment, survival rates were extended significantly when treated with AITC. Survival curves are presented based on three individual experiments.

Next, the nematodes were treated for five days with AITC (0 µM, 100 µM, 500 µM, and 1 mM) from L1 hatchees, transferred into S medium containing 200 µM juglone, and checked every hour for viability. When the nematodes were grown on 200 µM juglone alone, the survival rate decreased in a time-dependent manner, i.e., 60.5% (2-hour), 40.5% (4-hour), and 28.4% (6-hour), but the AITC treatment extended the survival rate significantly (Figure 5, Table 3).

**Table 3:** Protective effect of AITC on *C. elegans* against juglone.

		Survival %				
Concentration^a^	2 hour	4 hour	6 hour	Repeat	*p* value^b^	Total nematode
0 µM AITC	60.5%	40.5%	28.4%	3		190
100 µM AITC	70.2%	57.1%	44.9%	3	0.002	198
500 µM AITC	68.8%	62.9%	58.6%	3	0.000	186
1 mM AITC	76.0%	67.7%	67.7%	3	0.000	167

a, P values were calculated by log-rank test (compared with 0 µM AITC).

### AITC Also Induces UGT-13

**Figure 6:**
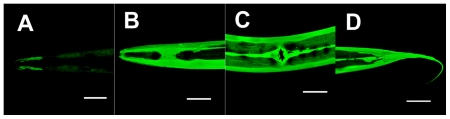
Expression patterns in MJCU050 {*kIs22 [ugt-13p::gfp, pDP#MM016B]* III}. Photographs were taken with laser-scanned fluorescence microscopy. (A) Control without acrylamide. Weak GFP signal was detected from head. (B, C, D) With 500 mg/L acrylamide. Strong GFP signals were detected from hypodermis. (B) Head region. (C) Vulval region. (D) Tail region. Scale bars, 50 µm.

**Figure 7:**
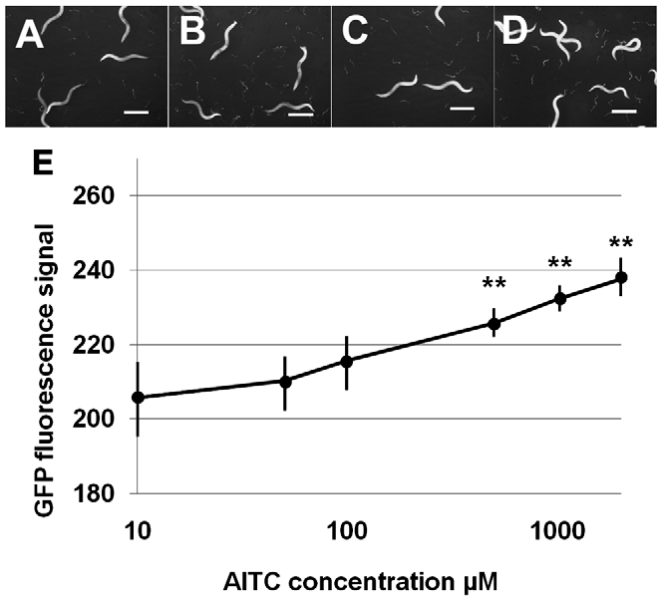
AITC induces UGT-13 expression. Fluorescence images of MJCU050 exposed to each AITC concentration from late L4 stage for 24 hours at 25°C. (A) Control 0 µM AITC. (B) 100 µM AITC. (C) 500 µM AITC. (D) 2 mM AITC. (E) GFP fluorescence signals were measured and plotted. GFP signals were increased at concentrations of 500 µM (Kruskal-Wallis test, ** *p*<0.005) in a dose-dependent manner. Scale bars, 500 µm.

As we obtained good evidence that AITC induced GST-4, a member of the phase II enzymes that play central roles for protection against endogenous and exogenous oxidative stresses, we next wanted to examine whether AITC could induce other phase II enzymes. For that task, we chose UGT-13, a member of another large phase II enzyme family of UDP glucuronosyl transferases. We constructed the new transgenic nematode MJCU050 {*kIs22 [ugt-13p::gfp, pDP#MM016B]*} possessing the *ugt-13* gene promoter (*ugt-13p*) fused with the *gfp* gene integrated in chromosome III. We confirmed that this nematode biosensor MJCU050, which emitted a residual constitutive level of the GFP signal similarly as did the GST biosensor, responded well against the harmful food substance acrylamide (Figure 6). When treated with AITC, MJCU050 started to express the GFP fluorescence signal over the control level from the 500 µM AITC level on (Figure 7).

### AITC Induces GST-4 and UGT-13 Expression via the Transcription Factor SKN-1

Previously we reported that acrylamide-induced GST-4 expression was partially regulated by the transcription factor SKN-1 [15]. To examine whether the GST-4 and UGT-13 expressions induced by AITC were also regulated under SKN-1 control, we performed knockdown experiments by feeding RNAi.

**Figure 8:**
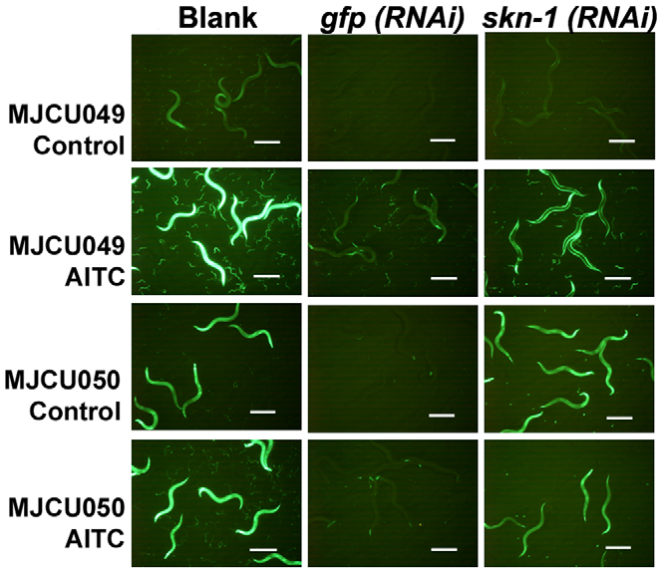
AITC induces GST-4 and UGT-13 expression via the transcription factor SKN-1. Although the residual amounts of both GST-4 and UGT-13 were observed to be expressed constitutively without AITC (Control), their expressions were induced well over the constitutive level after AITC treatment. Both constitutive and inducible expressions of GST-4 were suppressed by *gfp* and *skn-1 (RNAi)*. Inducible expression of UGT-13 was also suppressed by *gfp* and *skn-1 (RNAi)*, but constitutive expression of UGT-13 was suppressed by only *gfp (RNAi)*, but not by *skn-1 (RNAi)*. Inducible GST-4 expression in neither pharynx nor body-wall muscle was suppressed by *skn-1 (RNAi)* (Hasegawa et al., 2008). Inducible GST-4 expression in the pharynx was not suppressed by *gfp (RNAi)* indicating that RNAi, itself, does not work in the pharynx. Scale bars, 500 µm.

Whereas without AITC both MJCU049 and MJCU050 constitutively emitted a dim amount of the GFP signals, upon exposure to 2 mM AITC they each emitted much stronger GFP signals from the whole body (Figures 8, S1, S2). The knockdown of *gfp* by feeding RNAi inhibited the residual constitutive expression for both GST-4 and UGT-13, as expected. Although the RNAi feeding knockdown of *skn-1* prevented the constitutive GST-4 expression, it failed to prevent the constitutive UGT-13 expression, thus suggesting that such expression is not under SKN-1 control (Figure 8). SKN-1, however, did regulate the AITC-induced UGT-13 expression. We also found that SKN-1 regulated AITC-induced GST-4 expression except for that in the body-wall muscle.

## Discussion

Primarily because of improvements in public health and lifestyle, our life expectancy today is much longer than before. It means at the same time that we have a higher chance of suffering from age-related diseases as we live longer. Thus, we need to seek ways to improve the quality of our life, especially later in our life. One way to do so is to find foods or food ingredients that reduce the risks of suffering from cancer or age-related diseases. Inducers of phase II enzymes without adverse effects would be candidates for reducing such risks.

GSTs and UGTs comprise a large family of enzymes, which generally exist in every organism from bacteria to humans, and they are considered major players in the phase II detoxification of both endogenous oxidative stress products and exogenous electrophilic chemical compounds [9], [16], [17]. The *C. elegans* genome contains 52 *gst*- and 72 *ugt*-coding genes. The 52 *gst* genes belong to the Alpha, Sigma, Omega, Zeta, and Pi classes (WormBase, http://www.wormbase.org/), and their expression patterns were found to vary uniquely [15]. GST-4, which encodes a sigma class GST, was one of the most prominently up-regulated genes when the nematode was treated with the harmful food substance acrylamide [15], the food contaminant methylmercury (Hasegawa and Miwa, unpublished result), and the oxidative stressors paraquat and juglone [18], [19]. The GST-4 expression correlated with oxidative stress tolerance in a dose-dependent manner [19].

AITC, formed from glucosinolate, is an allelochemical compound having an isothiocyanate (ITC, –N = C = S) group, which is widely found among various cruciferous vegetables, such as broccoli, cabbage, and cauliflower [10], [20]. Isothiocyanates have been reported to induce several phase II enzymes *in vitro* and *in vivo* [11]–[13] and protect mammalian cultured cells against oxidative damage [7], [21], [22]. The nematode biosensor successfully evaluated AITC as being a very good GST-4 and UGT-13 inducer, suggesting that AITC might confer on the nematode resistance to oxidative stress. We could determine the optimal AITC concentration for the nematode biosensor; that is, AITC concentrations from 100 µM to 1 mM strongly induced both GST-4 and UGT-13 *in vivo* without causing adverse effects on either nematode fecundity or lifespan. The high concentration of 2 mM AITC, however, exerted a toxic effect on the nematodes, as it decreased both their fecundity and lifespan. Not only beneficial effects, but also toxic effects such as carcinogenicity and mutagenicity have been reported [23], [24]. High concentrations of isothiocyanates were reported to kill the potato cyst nematode *Globodera rostochiensis* and the eggs of the black vine weevil *Otiorhynchus sulcatus*, and their potential use as a pesticide for controlling nematodes or weevils in the soil was discussed [25], [26]. The mechanism of toxicity such as killing, however, remains to be investigated. AITC was also reported to elicit acute pain and neurogenic inflammation by activating TRPA1, a member of the mammalian transient receptor potential ion channel family [27], [28]. *C. elegans* TRPA-1, the ortholog of the mammalian TRPA1, was suggested to function in mechanosensation [29]. It is interesting to ask if TRPA-1 exerts a protective role against juglone or paraquat in response to AITC and/or if TRPA-1 is also under the SKN-1 regulation (Figures S3, S4).

With *C. elegans* as a monitoring organism, many synthetic and natural compounds that increased *C. elegans* lifespan have been discovered, including the anticonvulsants ethosuximide, trimethadione, and valproic acid, the antidepressant 3,3-diethyl-2-pyrrolidones [30]–[32], blueberry polyphenol [33], extracts of *Eleutherococcus senticosus* and *Rhodioa rosea* [34], and an extract of *Ginkgo biloba* [35], the last of which also increased survival rate against several stresses. Epigallocatechin gallate (EGCG) did not extend *C. elegans* lifespan but attenuated the age-related behavioral decline [36] and extended survival rate under heat stress and oxidative stress by inducing SOD-3 and HSP-16.2 [37]. Noticeably, EGCG induced *skn-1* mRNA [37], possibly suggesting that EGCG, like AITC in our present report, might have induced phase II enzymes, which consequently would have conferred oxidative stress tolerance on the nematode. Our biosensor would easily test this hypothesis.

In mammals, several phase II enzymes, like GST, are regulated by the bZIP transcriptional factor Nrf2 in response to oxidative or electrophilic stresses [8]. Without such stresses, Nrf2 is normally repressed by the oxidative stress sensor Keap1 [38], which binds and degrades Nrf2 through the ubiquitin-proteasome system. Under oxidative stress conditions, Nrf2 is released from Keap1 into the nucleus where it induces the expression of phase II enzymes [38]. In *C. elegans*, the transcription factor SKN-1 is found to function similarly to Nrf2 by inducing GCS-1 expression in response to oxidative stresses [39] and GST-4 expression in response to acrylamide [15]. And also in *C. elegans*, WDR-23, a *C. elegans* homolog of mammalian WDR23 and a WD40 repeat protein, seems to function similarly to the mammalian Keap1 [40] (Hasegawa and Miwa, unpublished results). Although without stresses MJCU049 and MJCU050 expressed a very low amount of a constitutive level for both GST-4 and UGT-13, treating them with AITC induced these enzymes well over the constitutive level. SKN-1 regulated both the constitutive and inductive expressions of GST-4; however, it did not regulate the constitutive expression of UGT-13. This result suggests that some factor other than SKN-1 should regulate the constitutive expression of UGT-13. The present nematode biosensor is not only a tool to evaluate and discover functional food ingredients but also would be a powerful genetic tool to dissect the GST and UGT expression pathways and to analyze their regulatory mechanisms (Hasegawa, Nonomura, Kondo, and Miwa, unpublished results).

Because the nematode biosensor we have established here takes direct advantage of its own sensing and responding systems against xenobiotic chemicals, we could apply it to (a) detect and evaluate harmful chemicals (15), (b) discover chemicals that ameliorate, reduce, or neutralize harmful chemicals [14], and (c) discover chemicals that improve our health or extend our longevity. As such is the case, the nematode biosensor serves as a handy, rapid, and inexpensive method not only to evaluate but also to discover chemicals, both harmful and useful, whether they are known or unknown, albeit an obvious caution is required when results so obtained are applied to humans.

## Materials and Methods

### Nematode Strains and Culturing

*C. elegans* var. Bristol, strain N2 [41] and *unc-119* (*ed3*) [42] were obtained from the *Caenorhabditis elegans* Genetics Center (University of Minnesota, Minnesota, Minneapolis, MN, USA). The nematodes were cultured and handled according to the procedures described by Brenner [41]. Allyl-isothiocyanate (AITC)-containing NGM plates were prepared as follows: 100 µL of AITC (Tokyo Chemical Industry, Tokyo, Japan) was diluted in 5 ml of 100% ethanol (Wako, Tokyo, Japan) to prepare the 200 mM stock solution. This was then serially diluted in 100% ethanol as described [43]. Fifty micro liters of each AITC concentration or ethanol as a control was added directly into a 6 cm Petri dish, and 5 mL of NGM agar was pipetted into the dish and immediately mixed by gentle swirling. All AITC plates were prepared two days before use and seeded with *Escherichia coli* OP50 one day before use.

### Nematode Biosensors

Because of their potent responses against several xenobiotics (acrylamide, methylmercury, and chloropropanols, Hasegawa and Miwa, unpublished results), we selected two phase II enzymes, UGT-13 and GST-4, and made their GFP reporter constructs for use as nematode biosensors. PCR was performed with KOD Plus DNA polymerase (TOYOBO, Osaka, Japan) on N2 genomic DNA. The PCR primers for *ugt-13* and *gst-4* were designed to amplify each predicted promoter upstream from each predicted start site; UGT-13For_SphI, 5′ – AAG GGG CAT GCA TTA TTA TGT TCC TAT TTC TTT TA – 3′, UGT-13pRev_BamHI, 5′ – CGG GAT CCC ATT GGG AAT TTT TCT TGA AAC AA – 3′, GST-4ForVer3_SalI, 5′ – GGG TCG ACT TTT GCA GAC TAA AAA TAA CTA CTC TG – 3′, GST-4pRev_BamHI, 5′ – CGG GAT CCC ATA ATT AGA ATT CAG TAA TTG AAT CG – 3′. PCR-amplified DNA fragments were digested with the appropriate restriction enzymes and then ligated into the *gfp* vector pPD95.77 (kindly provided by A. Fire, Stanford University) to obtain the reporter constructs. Each reporter construct (100 µg/mL) so obtained was co-injected with an equal concentration of *pDP#MM016B* into the gonadal arms of *unc-119* (ed3) adult hermaphrodites as described [44] to obtain MJCU020 {*kEx20 [ugt-13p::gfp, pDP#MM016B]*} and MJCU032 {*kEx32 [gst-4p::gfp, pDP#MM016B]*}. Each transgene extrachromosomal array was chromosomally integrated by the method of Mitani [45] and outcrossed two times with N2 to obtain MJCU049 {*kIs48 [gst-4p::gfp, pDP#MM016B]*} and MJCU050 {*kIs22 [ugt-13p::gfp, pDP#MM016B]*}. SNP (Single Nucleotide Polymorphism) -based mapping [46] located the fusion gene *gst-4p::gfp* of MJCU049 integrated in the linkage group V and, similarly, the fusion gene *ugt-13p::gfp* of MJCU050 in the linkage group III (data not shown). GFP expression patterns were observed with both a Nikon SMZ800 dissection microscope equipped with a fluorescence filter (GFP LP filter) and a ZEISS Axiovert 200 microscope equipped with a confocal laser-scanning module.

### GFP Signal Kinetics

Synchronized L1-stage transgenic nematodes were obtained by treating egg-containing adults with sodium hypochlorite [47] and allowed to grow on NGM plates seeded with *E. coli* OP50 at 20°C for 48 hours until the late L4 stage. About one hundred of these nematodes were collected and put onto each OP50-seeded 6 cm NGM plate, with each concentration of AITC or without it (control), and incubated at 25°C. After 24 hours of incubation, stereo photomicrographs of individual nematodes were taken with a Nikon SMZ800 equipped with a GFP fluorescence module. GFP fluorescence signals from the individual nematodes were measured from the photomicrographs with a nematode image analyzer “FL ver. 1.0.0.1” (http://www.vision.cs.chubu.ac.jp/fl1001/). Nonparametric one-way ANOVA (Excel Tokei 2006, SSRI, Tokyo, Japan) was used to determine the significance of differences in the mean GFP signal values.

### Brood Size Counting

All experiments were carried out at 20°C. Synchronized first larval stage (L1) nematodes (N2 strain) were transferred onto the bacterial-lawned NGM plates with AITC at various concentrations or without it (control) and incubated for two days. Single late L4 stage nematodes were then transferred onto the bacterial-lawned NGM spot plates with AITC at various concentrations or without it (control), and total numbers of fertilized eggs laid were counted by using a Nikon SMZ800. Nonparametric one-way ANOVA (Excel Tokei 2006, SSRI, Tokyo, Japan) was used to determine the significance of differences in the mean brood sizes.

### Lifespan Measurement

Synchronized L1 stage nematodes (N2 strain) were transferred onto the bacterial-lawned NGM plate with AITC at various concentrations or without it (control) and incubated for two days at 20°C. Two-day old (Late L4 stage) nematodes were then transferred onto 2.5 mg/L 5-fluoro-2-deoxyuridine (FdUrd, Sigma) -containing NGM plates seeded with *E. coli* OP50 with each AITC concentration or without it, and incubated at 25°C. This time point represented the first day of life-span analysis. Three-day old nematodes (five-days after synchronization) were again transferred onto the fresh AITC or control FdUrd plates and checked daily for viability by using a Nikon SMZ800. A nematode was considered dead when it no longer responded to light prodding with a platinum wire. Survival curves were analyzed by the Kaplan-Meier procedure, and significant differences between survival curves were calculated by the log-rank test with statistical software Excel Tokei 2006 (SSRI, Tokyo, Japan). All fresh AITC plates were prepared on the day before use.

### Stress Resistance Test

Synchronized three-day old (five days after synchronization) adults treated with each AITC concentration were prepared as described above. For the oxidative stress resistance assay, AITC-treated three-day old nematodes (five days after hatching) were transferred onto NGM plates containing 10 mM paraquat (Wako, Osaka, Japan), or into wells of a 48-well plate containing S medium [47] and 200 µM juglone (Calbiochem, Darmstadt, Germany) in a total volume of 200 µL per well. Both assays were incubated at 20°C and were checked for viability daily (for the paraquat test) or every hour (for the juglone test) by using a Nikon SMZ800.

### RNAi

Gene fragments of *skn-1* or *gfp* were prepared by PCR amplification of *C. elegans* N2 cDNA or plasmid vector pPD95.77 with the following primers: Ceskn1EcoRI_For, 5′ – GGA ATT CGG CCA ATC CAA ATA TGA TTA TCC A – 3′; Ceskn1EcoRI_Rev, 5′ – GGA ATT CGG GCA GCA ACC TTG TTC TTT CCG – 3′ or GFPFor_EcoRI, 5′ – GGG AAT TCA CTG GAG TTG TCC CAA TTC TT – 3′; GFPRev_EcoRI, 5′ – GGG AAT TCA TCC ATG CCA TGT GTA ATC CC – 3′. Both of the *skn-1*- or *gfp*-derived PCR fragments were digested with *Eco*RI and cloned into the *Eco*RI restriction site of the RNAi vector pPD129.36 (Kindly provided by Fire, A., Stanford University). The PCR fragment-ligated plasmids or the blank vector pPD129.36 were used to transform *E. coli* HT115 [48].

For RNAi experiments, synchronized L1-stage transgenic nematodes were first grown for 48 hours at 20°C with NGM (containing 50 µg/ml ampicillin and 12.5 µg/ml tetracycline) plates seeded with *E. coli* HT115 transformed with each different RNAi plasmid. Thereafter, the nematodes were collected and transferred onto NGM plates, with or without 2 mM AITC, seeded with each different *E. coli* HT115 RNAi bacteria, and grown for 24 hours at 20°C. The nematodes were observed for GFP expression by using a Nikon SMZ800 dissection microscope equipped with a GFP fluorescence filter.

## Supporting Information

Figure S1 GFP fluorescence signals were measured. GST-4 expression was induced with 2 mM AITC (p<0.005) over its constitutive expression. Both constitutive and inducible GST-4 expressions were suppressed by skn-1 (RNAi) (p<0.005) except for in the body-wall muscle and pharynx. For a more detailed explanation, refer to the Figure 8 legend. Significance of differences in the mean GFP signal values was calculated by nonparametric one-way ANOVA.
(0.10 MB TIF)

Figure S2 GFP fluorescence signals were measured. UGT-13 expression was induced with 2 mM AITC (p<0.005) over its constitutive expression. Inducible UGT-13 expression was suppressed by skn-1 (RNAi) (p<0.005), but constitutive UGT-13 expression was not. For a more detailed explanation, refer to the Figure 8 legend. Significance of differences in the mean GFP signal values was calculated by nonparametric one-way ANOVA.
(0.10 MB TIF)

Figure S3 AITC conferred resistance against 200 µM juglone, but this resistance essentially disappeared by skn-1 (RNAi), suggesting that the AITC-induced protection observed arose only through the AITC-activated skn-1 activity. Animals were treated with 1 mM AITC with or without skn-1 (RNAi) from L1 stage; and juglone resistance assay was performed as described in Materials and Methods. Survival curves (based on three individual experiments) were analyzed by the Kaplan-Meier procedure, and significant differences between survival curves were calculated by the log-rank test.
(0.10 MB TIF)

Figure S4 AITC conferred resistance against 10 mM paraquat. Not only this resistance disappeared by skn-1 (RNAi) but resistance reduced below even that for controls, suggesting that SKN-1 has a protective role against paraquat without AITC. Animals were treated with 1 mM AITC with or without skn-1 (RNAi) from L1 stage, and the paraquat resistance assay was performed as described in Materials and Methods. Survival curves (based on two individual experiments) were analyzed by the Kaplan-Meier procedure, and significant differences between survival curves were calculated by the log-rank test.
(0.10 MB TIF)
