# Supplementary figures and images for: Allyl Isothiocyanate that Induces GST and UGT Expression Confers Oxidative Stress Resistance on C. elegans, as Demonstrated by Nematode Biosensor
Source: PLoS One. 2010 Feb 17;5(2):e9267. doi: 10.1371/journal.pone.0009267 (PMC2822842; doi:10.1371/journal.pone.0009267)

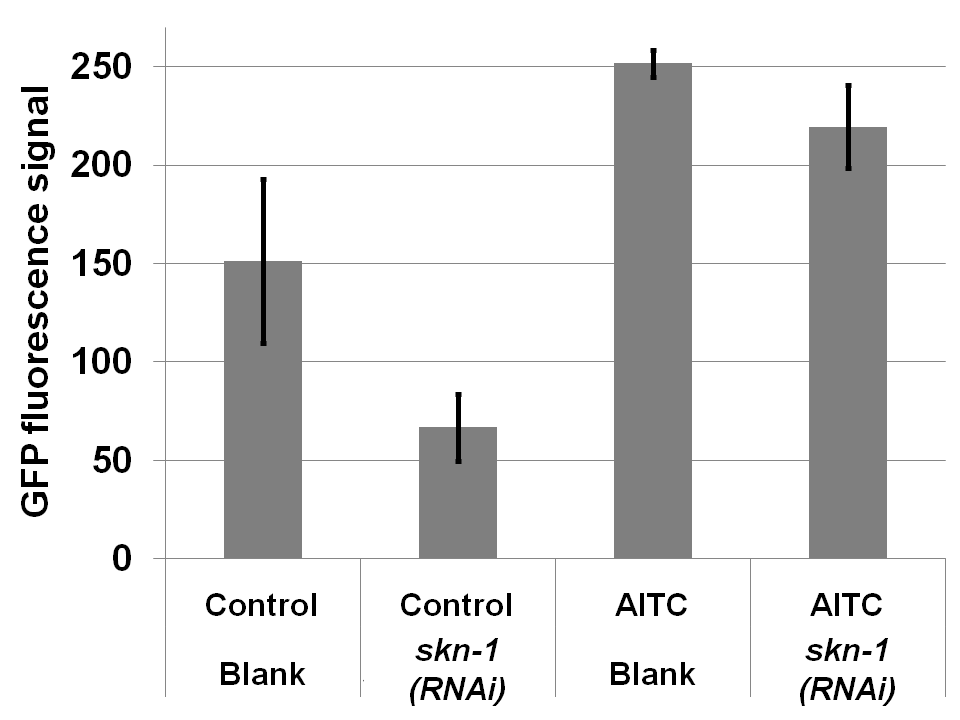

Supplement: Figure S1 — GFP fluorescence signals were measured. GST-4 expression was induced with 2 mM AITC (p<0.005) over its constitutive expression. Both constitutive and inducible GST-4 expressions were suppressed by skn-1 (RNAi) (p<0.005) except for in the body-wall muscle and pharynx. For a more detailed explanation, refer to the Figure 8 legend. Significance of differences in the mean GFP signal values was calculated by nonparametric one-way ANOVA. (0.10 MB TIF) [file pone.0009267.s001.tif]

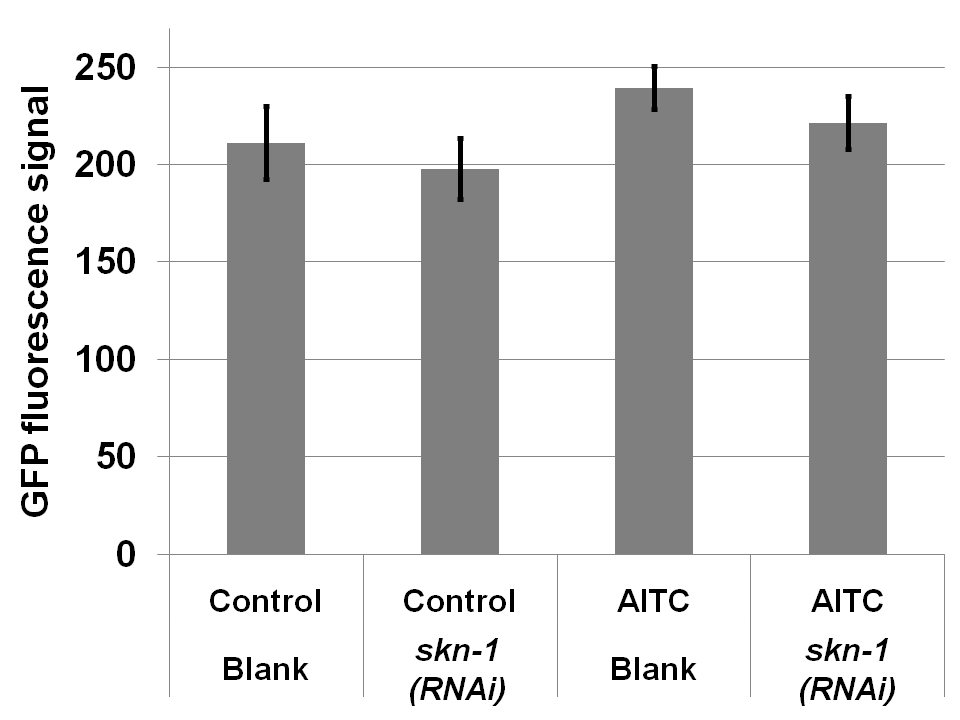

Supplement: Figure S2 — GFP fluorescence signals were measured. UGT-13 expression was induced with 2 mM AITC (p<0.005) over its constitutive expression. Inducible UGT-13 expression was suppressed by skn-1 (RNAi) (p<0.005), but constitutive UGT-13 expression was not. For a more detailed explanation, refer to the Figure 8 legend. Significance of differences in the mean GFP signal values was calculated by nonparametric one-way ANOVA. (0.10 MB TIF) [file pone.0009267.s002.tif]

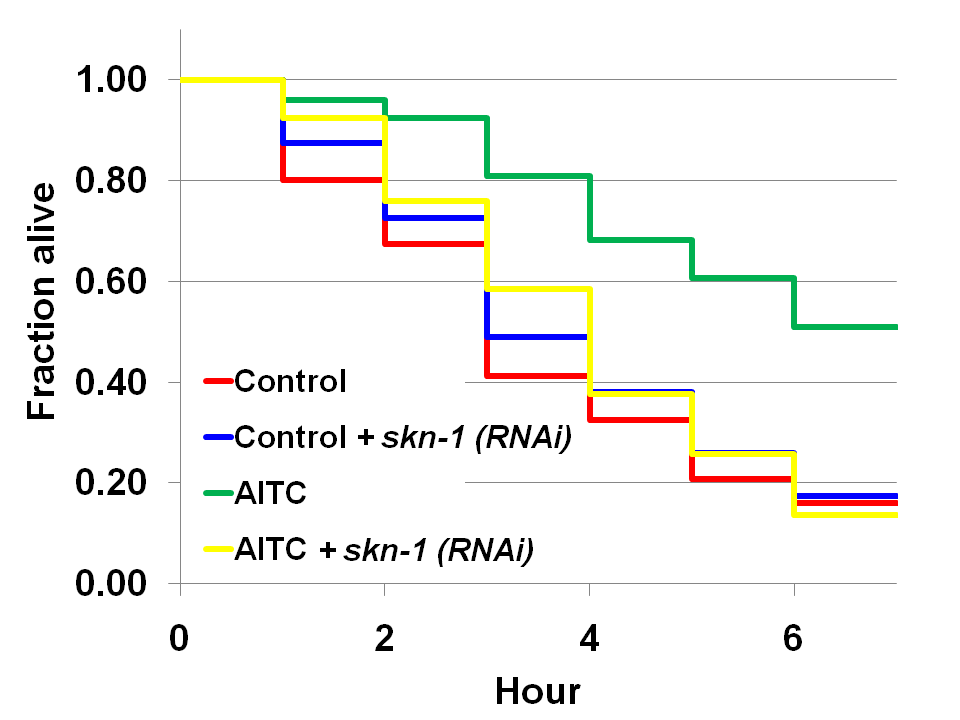

Supplement: Figure S3 — AITC conferred resistance against 200 µM juglone, but this resistance essentially disappeared by skn-1 (RNAi), suggesting that the AITC-induced protection observed arose only through the AITC-activated skn-1 activity. Animals were treated with 1 mM AITC with or without skn-1 (RNAi) from L1 stage; and juglone resistance assay was performed as described in Materials and Methods. Survival curves (based on three individual experiments) were analyzed by the Kaplan-Meier procedure, and significant differences between survival curves were calculated by the log-rank test. (0.10 MB TIF) [file pone.0009267.s003.tif]

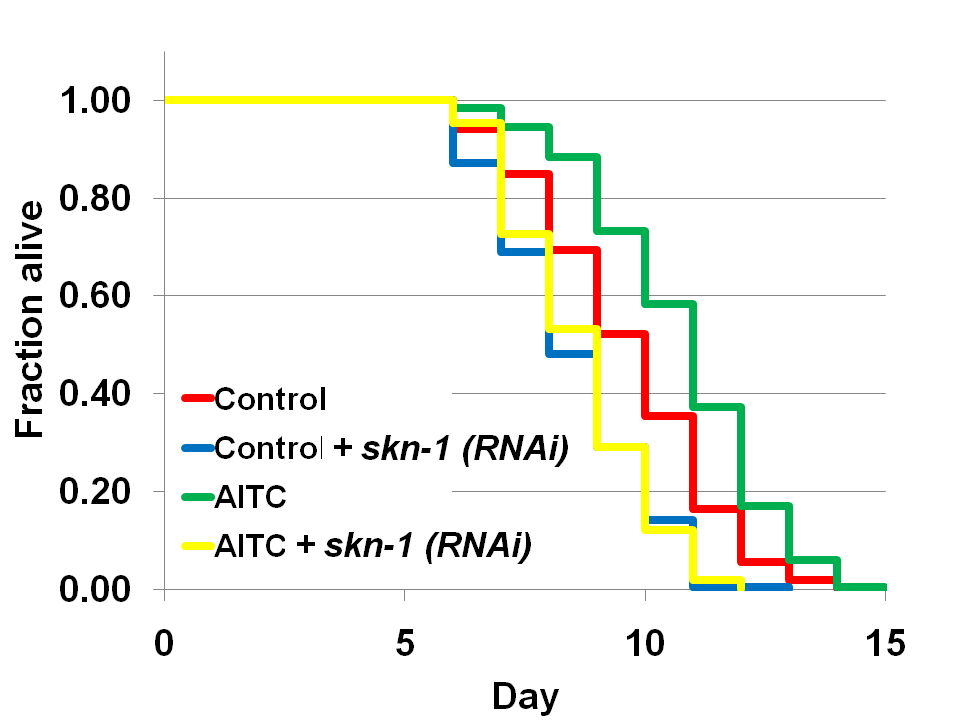

Supplement: Figure S4 — AITC conferred resistance against 10 mM paraquat. Not only this resistance disappeared by skn-1 (RNAi) but resistance reduced below even that for controls, suggesting that SKN-1 has a protective role against paraquat without AITC. Animals were treated with 1 mM AITC with or without skn-1 (RNAi) from L1 stage, and the paraquat resistance assay was performed as described in Materials and Methods. Survival curves (based on two individual experiments) were analyzed by the Kaplan-Meier procedure, and significant differences between survival curves were calculated by the log-rank test. (0.10 MB TIF) [file pone.0009267.s004.tif]
